# New Percepts via Mental Imagery?

**DOI:** 10.3389/fpsyg.2012.00360

**Published:** 2012-10-02

**Authors:** Fred W. Mast, Elisa M. Tartaglia, Michael H. Herzog

**Affiliations:** ^1^Department of Psychology, University of BernBern, Switzerland; ^2^Center for Cognition, Learning and Memory, University of BernBern, Switzerland; ^3^Laboratory of Psychophysics, Brain and Mind Institute, Ecole Polytechnique Fédérale de Lausanne (EPFL)Lausanne, Switzerland; ^4^Laboratory of Neurophysics and Physiology, UMR 8119 CNRS, Université René DescartesParis, France

**Keywords:** ambiguous figure, bisection, combinatorics, interpolation, mental rotation, top-down processing, perceptual learning

## Abstract

We are able to extract detailed information from mental images that we were not explicitly aware of during encoding. For example, we can discover a new figure when we rotate a previously seen image in our mind. However, such discoveries are not “really” new but just new “interpretations.” In two recent publications, we have shown that mental imagery can lead to perceptual learning (Tartaglia et al., 2009, 2012). Observers imagined the central line of a bisection stimulus for thousands of trials. This training enabled observers to perceive bisection offsets that were invisible before training. Hence, it seems that perceptual learning via mental imagery leads to new percepts. We will argue, however, that these new percepts can occur only within “known” models. In this sense, perceptual learning via mental imagery exceeds new discoveries in mental images. Still, the effects of mental imagery on perceptual learning are limited. Only perception can lead to really new perceptual experience.

Imagination is more important than knowledge. For knowledge is limited, whereas imagination embraces the entire world, stimulating progress, giving birth to evolution.

(Albert Einstein, 1929)

## Mental Imagery

Mental imagery is usually referred to as seeing with the mind’s eye. We are able to create images in our mind despite the absence of appropriate sensory stimulation. Mental images are usually described introspectively, using a sensory terminology such as form, color, spatial extent, and so forth. Even though introspection does not necessarily allow for drawing conclusions about the underlying mechanisms recent research has shown that people have a good metacognitive understanding of the vividness of their mental images (Pearson and Tong, 2011).

The mechanisms that underlie mental imagery have become the topic of the “Imagery Debate” (see for example Kosslyn et al., 2003; Pylyshyn, 2003). Cognitive psychologists have carried out a large amount of behavioral experiments to tap into the mechanisms that underlie mental imagery, and the results provide compelling evidence that mental imagery shares common mechanisms with visual perception. For example, it has been shown that mental images preserve spatial distances. In experiments on image scanning, participants had to first memorize visual information shown in a picture (e.g., a map of an island) and later answer questions about landmarks (Kosslyn et al., 1978). Interestingly, response times were proportional to the actual distances between the landmarks. It took participants more time to scan longer distances. Hence, participants were able to extract metric distance information from memory. Yet other experiments have shown that participants are well able to judge and compare distances in mental images. For example, when participants indicate whether a previously seen object is higher than wide (e.g., a picture of a sailboat) they are able to extract this information from mental images even though they did not attend to the spatial dimensions when they encoded the visual stimulus (Kosslyn et al., 1995). It has been argued that participants learned the stimuli beforehand, and that tacit knowledge about the purpose of the experiment could have led to the findings. Finke and Pinker (1982) showed participants an array of dots. After the dots disappeared, an arrow was presented and participants decided whether or not the arrow pointed to one of the dots they just saw. Response times increased linearly with increasing distance between the arrow and the target dot. It has been proposed that attentional crowding could account for the distance effect, leading to more difficult discriminations for further distances (Pylyshyn, 2002). However, this possible explanation has been ruled out by Dror and Kosslyn (1993) who conducted a modified version of Finke and Pinker’s experiment. They replaced dots by black (3) and white (17) squares which were arranged in a square-like configuration (six squares on each side). The task remained the same as in Finke and Pinker’s experiment but the distance between the black squares was chosen so that it exceeded the distance known to produce attentional crowding (Intriligator and Cavanagh, 2001). As expected, Dror and Kosslyn (1993) demonstrated that response times increased with increasing distance between the arrow and the square. Hence, their results confirmed that mental images embody metric properties even when no spatial relationships are encoded explicitly during the presentation of the stimulus. Yet other evidence comes from neuroimaging studies showing a large overlap in activated areas during mental imagery and visual perception, including early visual cortex (e.g., Slotnick et al., 2005). Activation in the latter has been found when participants were engaged in mental imagery tasks that required the extraction of high-resolution visual information. In addition, TMS over the occipital cortex disrupted performance in those tasks (Kosslyn et al., 1999). Thus, there is compelling evidence for commonalities between mental imagery and perception. However, it has to be pointed out that there are also differences, and, for example, only rarely do we confuse images with percepts. The discovery of new information is yet another conceivable difference between mental imagery and perception. When we inspect with our eyes a visual scene new information is picked up continuously. We discover more and more details that are part of the visual scene. It has been argued that new discoveries cannot be made in mental imagery (Chambers and Reisberg, 1985, 1992). A mental image contains nothing new besides the information the observer is aware of when generating the image. Ambiguous figures, for example, demonstrate compellingly that the perceptual interpretation of the same visual stimulus can switch from one explanation to another. Observers usually do not notice the alternative perceptual explanation when first viewing the ambiguous figure. Obviously, new discoveries occur in perception. What about mental images? Are people able to discover new information in mental images? Mast and Kosslyn (2002) used an ambiguous figure in which the alternative perceptual explanation was upside down (young lady, old lady). It was almost impossible to discover the second explanation (e.g., young lady upside down) accidentally during encoding. However, roughly 50% of participants were able to discover in their mental image a new upright interpretation after having mentally rotated the image by 180°. None of the participants was aware about the alternative explanation when they learned the image (in one orientation only). Hence, observers discovered a new interpretation that they were not aware of when encoding the image. Thus, the ability to extract new interpretations is not bound to the process of perception as it can occur just as well during mental imagery. The ability to discover new interpretations in images is not the crucial difference between imagery and perception. Mental images are not interpreted entities as claimed by Fodor (1975) and they are not tied to their initial interpretation assigned during image generation. Indeed, participants were not aware of the second interpretation in the image even though all the information was available in the stimulus they encoded. Hence, only a new interpretation but no new information was discovered. Here is another example. Imagine a letter “D” and rotate it counterclockwise by 90°, and then take the letter “J” and attach it to the rotated “D” so that the upper end of the “J” is attached exactly at the middle of the horizontal line in the rotated “D.” What does it look like? An umbrella. The umbrella does not make part of its individual constituents (D, J) but still people are able to extract the shape from the configuration created in imagery. Indeed, a major strength of mental imagery is the ability to recombine information, thus, going beyond perceptual experience. Therefore, mental imagery serves an important function in creative thinking. However, the detection of emergent new shapes in imagery still remains in the realm of potential interpretations.

This article focuses on the relation between mental imagery and perceptual learning. At first glance, these topics appear somewhat unrelated but we were able to demonstrate in a series of experiments the existence of perceptual learning via mental imagery. Perceptual learning without perceptual input opens new ways of using mental imagery in learning paradigms, thus challenging classical views on perceptual learning. The fact that mental imagery and visual perception share – at least in part – the same mechanisms extends to learning and memory. We gathered evidence that learning in imagery transfers to perceptual performance (Tartaglia et al., 2009, 2012). The next paragraph will provide an overview of perceptual learning, and focus on the implications that learning via mental imagery has on the current understanding of mental imagery and perception alike.

## Perceptual Learning

**Figure 1:**
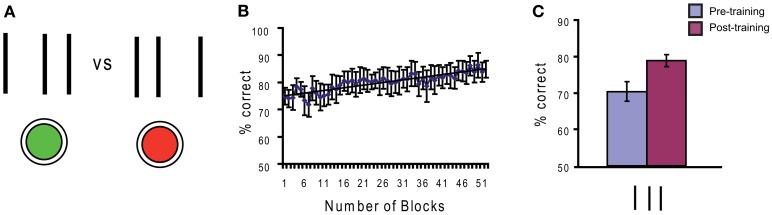
**(A)** In line bisection, a spatial interval, delineated by two outer lines, is bisected in two unequal components by a central line. The task of the observer is to judge whether the central line is closer to the left or right outer line. Observers are asked to give their response by using one of two buttons. **(B)** Performance improves with training, i.e., observers become able to discriminate offset sizes indiscriminable before training. **(C)** Another way of measuring improvements of performance is to compare observers’ performance before (“pre-training”) and after (“post-training”) the training phase. Post-training baselines are significantly higher than pre-training baselines.

Perceptual learning is learning to perceive. Sommeliers can taste not only the grape of a wine but often the year of its production and vineyard. Years of training make the master. In the laboratory, training improves basic visual skills including the discrimination of motion direction (Ball and Sekuler, 1987), the detection of Gabor gratings (Sowden et al., 2002), stereoacuity (Fendick and Westheimer, 1983; Kumar and Glaser, 1993), line orientation judgments (Vogels and Orban, 1985), texture discrimination (Karni and Sagi, 1991; Pourtois et al., 2008) and the discrimination of fine spatial features, such as in vernier acuity (Fahle and Edelman, 1993). Another example are bisection stimuli where a central line is closer to one of two outer lines (Figure 1). Observers indicate the offset direction (to the left or to the right). Training can strongly improve offset discrimination (Figure 1).

Without phylogenetic or ontogenetic perceptual learning, there is no perception (Herzog and Esfeld, 2009). Kittens that were raised in an environment missing horizontal lines during a critical period were blind for the horizontal orientation for the rest of their entire life (Blakemore and Cooper, 1970; Hirsh and Spinelli, 1970). In this respect, perceptual learning is often thought to be the most basic type of learning.

The mechanisms that underlie perceptual learning are controversially debated. However, there is clear consensus that perceptual learning is driven by the repeated exposure to the stimuli. Only repeated wine tastings make a good sommelier. On the neural level, the presentation of a stimulus leads to changes of synaptic weights which may, for example, fine tune receptive fields (e.g., Fahle, 2005) or enhance gating to decision stages (e.g., Herzog and Fahle, 1998; Lu and Dosher, 2004). Top-down aspects, such as attention, may be crucial in perceptual learning, but they are only modifying, but not driving the synaptic changes. For example, attention selects aspects of the stimuli to be learned, but the learning itself is driven by the stimuli.

In all mathematical models of perceptual learning, there is a stimulus related term essential for learning. In unsupervised learning algorithms, as for example in Hebbian learning, the synaptic changes Δ*w_j_* depend only on the concurrent activation of neurons:

$$\Delta w_{j}=\eta \cdot y^{\mu }x_{j}^{\mu }$$

where $x_{j}^{\mu }$ is the activity of the *j*-th input neuron in response to stimulus μ and $y_{j}^{\mu }$ is the activity of the output neuron in response to the same stimulus (η is the learning rate); $y_{j}^{\mu }$ is the weighted sum of the input $y_{j}^{\mu }=\Sigma w_{j}\;*x_{j}^{\mu }$ and hence fully determined by the actual input and the synaptic weights *w_j_*. If no stimuli are presented *x_j_* = 0, for all *j*, i.e., there is no activation of the input layer and hence no learning since Δ*w_j_* = 0, for all *j*. Hence, no perceptual learning is expected in the absence of stimulus presentation. Similar considerations hold for other types of neural networks of perceptual learning.

**Figure 2:**
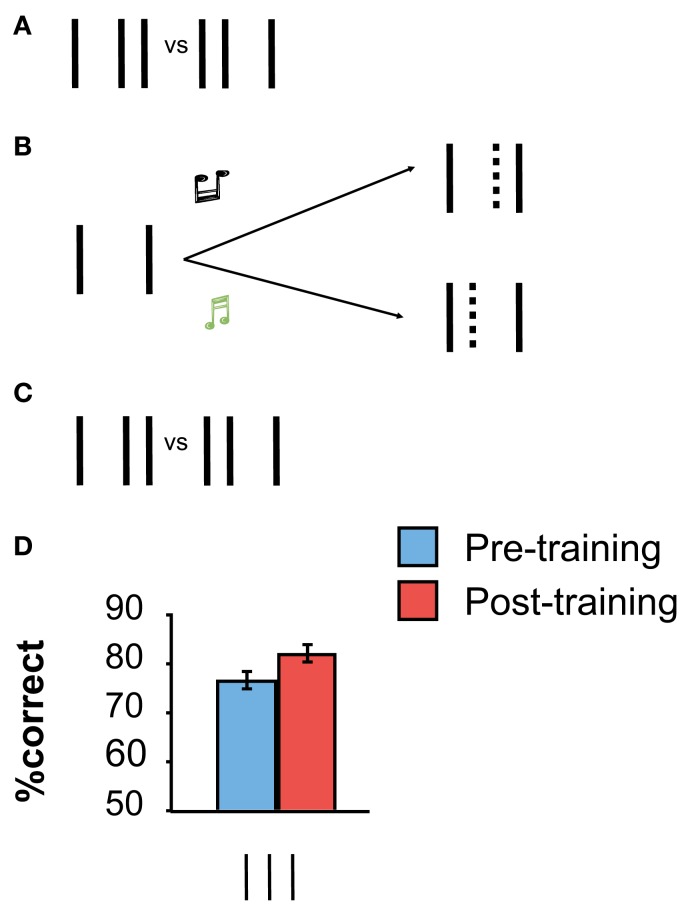
**(A,C)** Pre- and Post-training baseline measurements with the “proper” vertical bisection stimulus. **(B)** Imagery training. Only the two outer lines of the bisection stimulus are presented. Observers are asked to imagine the absent central line to be offset either to the left or to the right, depending on a tone, and to push buttons accordingly. We asked observers to imagine the smallest possible offset; the imagined line is indicated by the dashed line (which was not shown in the actual display). **(D)** Performance improved significantly through training. The error bars indicate the standard error of the mean (SEM) for nine observers. Adapted from Tartaglia et al. (2009).

However, contrary to all previous thinking in perceptual learning research, our recent experiments showed that perceptual learning can occur in the absence of (proper) stimulus presentation when participants imagine the missing perceptual information (Tartaglia et al., 2009, 2012). For example, we used a modified bisection stimulus in which only the outer lines were presented during training (Figure 2). Observers were asked to imagine the center line to be offset either to the left or right depending on a tone provided in addition (a high tone was associated to the right offset, a low tone to the left one). Hence, the physical stimulus was always the same in all trials. There was “nothing” to learn from the stimulus itself. Still, performance improved during mental imagery, as determined by pre- and post-training measurements in which the proper bisection stimulus was presented (Figure 2). Hence, mental imagery is sufficient to enable perceptual learning.

**Figure 3:**
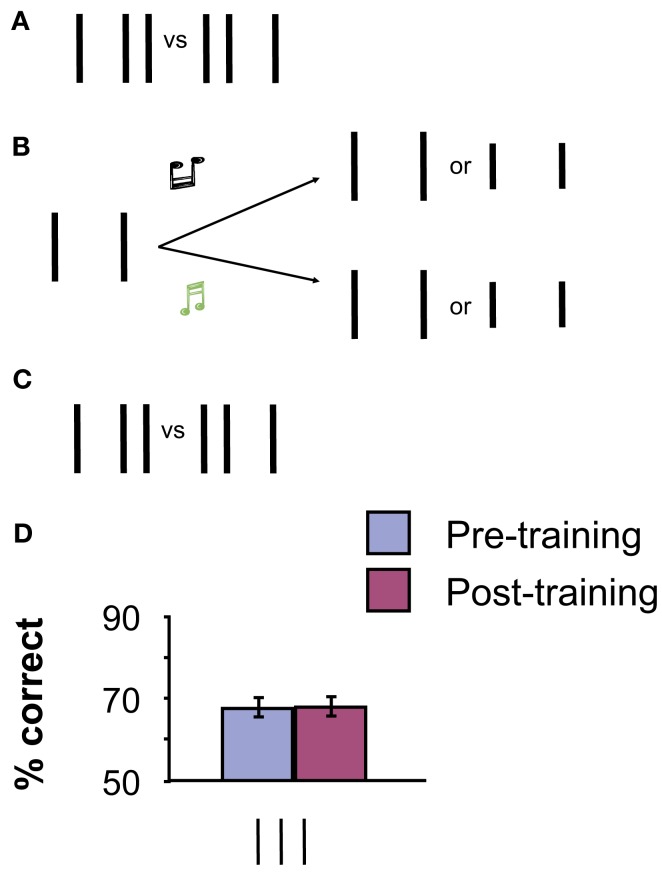
**(A,C)** Pre and Post-training baseline measurements with the proper vertical bisection stimulus. **(B)** Counting lines. Only the two outer lines were presented. Observers were asked to press the right button when a high-frequency tone was presented and the left button for a low-frequency tone. In addition, observers were asked to count the number of trials with shorter lines (0, 1, or 2 out of 80 trials per block). No imagery was involved in this experiment. **(D)** No significant improvement of performance occurred through training for the vertical bisection. The error bars indicate the standard error of the mean (SEM) for six observers. Adapted from Tartaglia et al. (2009).

The improvements during training are not caused by the pre- and post-measurements with the full bisection stimulus because measurements without training did not yield any performance gains (see Tartaglia et al., 2009). But is it really mental imagery that improves performance in perceptual learning? Unspecific effects such as better coping with attention and improved decision making need to be controlled for. We performed a control experiment with (almost) the same stimuli but without imagery during training. During the training phase, again only the two vertical outer lines of the bisection were presented, together with the two different tones. Observers had to press the right button when a high-frequency tone was presented and the left button when a low-frequency tone was presented. We did not ask observers to imagine the central line of the bisection stimulus. To ensure observers’ attention to the stimuli, the outer lines were only half as long as normal in a few trials (0, 1, or 2 within a block of 80 trials). At the end of each block, observers were required to report the number of trials with shorter lines. The physical stimulation was identical to the imagery experiment (except for the 0–2 lines deviating per block; Figure 3). Performance did not improve in this condition (Figure 3). Hence, it is the mental imagery training that leads to perceptual learning. Further experiments demonstrated perceptual learning via mental imagery for Gabor and motion stimuli (Tartaglia et al., 2009, 2012).

Interestingly, perceptual learning via motion imagery improves coherent motion discrimination of moving dots when a blank screen is presented. However, no improvement occurs when, instead of a blank screen, randomly moving dots are presented during training (Tartaglia et al., 2012). Contrary to motion stimuli perceptual learning in the bisection task requires the presence of the two outer lines during training. Without the lines, performance does not improve. In mental imagery studies with static stimuli, it is often the case that a perceptual reference is needed (see the role of perceptual assistance shown in other studies, e.g., Intons-Peterson, 1981; Mast et al., 1999). Future research will better define the conditions under which mental imagery training will unfold its impact on perceptual learning. Taken together, it is possible to learn to see things that were not visible before imagery training. Thus, is perceptual learning via mental imagery truly creating new percepts? We will argue: yes and no.

## Combinatorics

**Figure 4:**
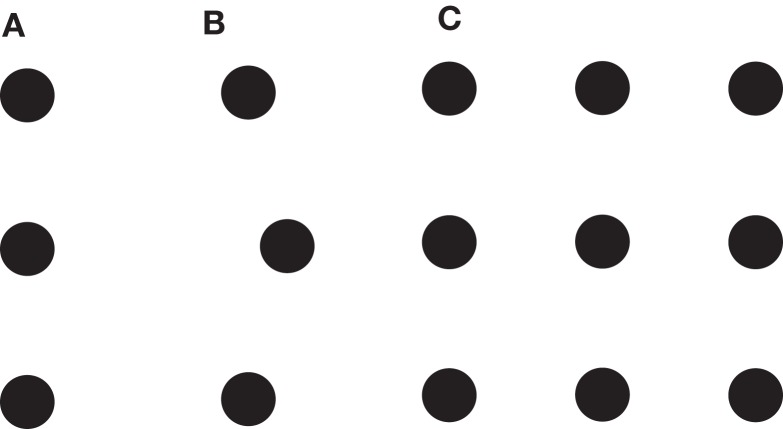
***Th*e *dot world***. **(A)** Three dots are presented that appear to be aligned. **(B)** After extended training, observers can easily discriminate the offset direction of the center dot. Perceptual learning has made spatial differences visible that were not visible before. **(C)** Nine dots are presented. In this display, $\left(\begin{array}{c} 9\\ 3 \end{array}\right)=84$ tasks with positional changes of three dots can be defined (the other six dots constitute the background). In general, 2^9^ tasks can be defined for 9 dots. In displays with n dots, hence, 2^n^ tasks can be defined leading to 2^n^ objects that one can potentially learn to perceive. $\left(\begin{array}{c} n\\ k \end{array}\right)=\frac{n!}{k\left(n-k\right)!}$ with *n*! = 1*2*3*… *n. From Herzog and Esfeld (2009).

In Figures 4A,B a 3-dot vernier is shown for which training improves performance (Fahle and Morgan, 1996). In Figure 4C, a set of nine dots is shown for which 84 3-dot vernier tasks can be defined and trained. Because of the specificity of perceptual learning, we expect no or very little transfer between tasks. Also 2 or 8-dot vernier tasks can be defined. In general, 256 vernier tasks can be defined by the 9-dot display. For 52 dots, there are more tasks than all the milliseconds in the universe that passed by since big bang. Hence, very small sets of elements can create very large combinatorial spaces.

We propose that mental imagery operates within such combinatorial spaces. The landmarks of the island that were memorized in the Kosslyn et al. (1978) experiment span a combinatorial space. Imagery does not add any new basic elements (e.g., a light tower) but computes, for example, second order aspects such as path lengths. In this sense, imagery does not create anything new. It just makes implicit aspects explicit. However, combinatorial spaces are large, often virtually infinite. Hence, the majority of combinatorial facts will remain unknown because only a small margin can be computed. In this sense, imagery produces new knowledge that may be unique to a certain person at a given moment in time.

On the first glance, it may appear as if perceptual learning via mental imagery can lead to really new percepts, as in the bisection stimulus where participants learn to perceive offsets that were not visible before. However, as mentioned above, without perceptual assistance provided by the outer lines, mental imagery training did not lead to perceptual learning. It seems that perceptual learning via mental imagery occurs within a model consisting of the two outer lines and the metric space in between. It is possible that the brain learns to better read out information from coding the center line. Hence, perceptual learning via mental imagery creates new percepts. However, these basic percepts are not so much different from detecting a new face in a mental image that was not visible during encoding. Still, all these percepts are new in the virtually infinite combinatorial space of things that can be learned and perceived. Once we have made the perceptual experience new knowledge can emerge from the combinatorial space. Really new percepts, however, are based on experience and they cannot be traced back to previous visual percepts. At this point, however, we enter the realm of philosophy and, admittedly, the definition of what a new percept is remains a debatable issue.

## Conclusion

Mental imagery can create new percepts via perceptual learning. The examples we summarized here can be explained by mental imagery relying on a generating model combining and changing past percepts. This would allow for creating infinite numbers of new percepts within vast combinatorial spaces. We suggest that only perception can generate really new percepts in the sense of model free extrapolation.

## Conflict of Interest Statement

The authors declare that the research was conducted in the absence of any commercial or financial relationships that could be construed as a potential conflict of interest.

## References

[B1] Ball K. Sekuler R. (1987). Direction-specific improvement in motion discrimination. Vision Res. 27, 953–965 10.1016/0042-6989(87)90011-3 3660656

[B2] Blakemore C. Cooper G. F. (1970). Visual experience modifies distribution of horizontally and vertically oriented receptive fields in cats. Science 168, 869–871 10.1126/science.168.3933.869 5444065

[B3] Chambers D. Reisberg D. (1985). Can mental images be ambiguous? J. Exp. Psychol. Hum. Percept. Perform. 11, 317–328 10.1037/0096-1523.11.3.317

[B4] Chambers D. Reisberg D. (1992). What an image depicts depends on what an image means. Cogn. Psychol. 24, 145–174 10.1016/0010-0285(92)90006-N 1582171

[B5] Dror I. Kosslyn S. M. (1993). Mental imagery and aging. Psychol. Aging 9, 90–102 10.1037/0882-7974.9.1.90 8185873

[B6] Einstein A. (1929). What Life Means to Einstein. Saturday Evening Post, 26, October.

[B7] Fahle M. (2005). Perceptual learning: specificity versus generalization. Curr. Opin. Neurobiol. 15, 154–160 10.1016/j.conb.2005.03.010 15831396

[B8] Fahle M. Edelman S. (1993). Long-term learning in vernier acuity: effects of stimulus orientation, range and of feedback. Vision Res. 33, 397–412 10.1016/0042-6989(93)90094-D 8447110

[B9] Fahle M. Morgan M. (1996). No transfer of perceptual learning between similar stimuli in the same retinal position. Curr. Biol. 6, 292–297 10.1016/S0960-9822(02)00479-7 8805246

[B10] Fendick A. Westheimer G. (1983). Effect of practice and the separation of test targets on foveal and peripheral stereoacuity. Vision Res. 23, 145–150 10.1016/0042-6989(83)90137-2 6868389

[B11] Finke R. A. Pinker S. (1982). Spontaneous imagery scanning in mental extrapolation. J. Exp. Psychol. Learn Mem. Cogn. 8, 142–147 10.1037/0278-7393.8.2.142 6210748

[B12] Fodor J. A. (1975). The Language of Thought. New York: Crowell

[B13] Herzog M. H. Esfeld M. (2009). How the mind constitutes itself through perceptual learning. Learn. Percept. 1, 147–154 10.1556/LP.1.2009.1.11

[B14] Herzog M. H. Fahle M. (1998). Modeling perceptual learning: difficulties and how they can be overcome. Biol. Cybern. 78, 107–117 10.1007/s004220050418 9525037

[B15] Hirsh H. V. B. Spinelli D. N. (1970). Development of the brain depends on the visual environment. Nature 228, 477–478 10.1038/228057a0 5482506

[B16] Intons-Peterson M. J. (1981). Constructing and using unusual and common images. J. Exp. Psychol. Hum. Learn. 7, 133–144 10.1037/0278-7393.7.2.133

[B17] Intriligator J. Cavanagh P. (2001). The spatial resolution of visual attention. Cogn. Psychol. 43, 171–216 10.1006/cogp.2001.0755 11689021

[B18] Karni A. Sagi D. (1991). Where practice makes perfect in texture discrimination: evidence for primary visual cortex plasticity. Proc. Natl. Acad. Sci. U.S.A. 88, 4966–4970 10.1073/pnas.88.11.4966 2052578 PMC51788

[B19] Kosslyn S. M. Ball T. M. Reiser B. J. (1978). Visual images preserve metric spatial information: evidence from studies of image scanning. J. Exp. Psychol. Hum. Percept. Perform. 4, 47–60 10.1037/0096-1523.4.1.47 627850

[B20] Kosslyn S. M. Ganis G. Thompson W. L. (2003). Mental imagery: against the nihilistic hypothesis. Trends Cogn. Sci. (Regul. Ed.) 7, 109–111 10.1016/S1364-6613(03)00025-1 12639690

[B21] Kosslyn S. M. Pascual-Leone A. Felician O. Camposano S. Keenan J. P. Thompson W. L. Ganis G. Sukel K. E. Alpert N. M. (1999). The role of area 17 in visual imagery: convergent evidence from PET and rTMS. Science 284, 167–170 10.1126/science.284.5411.167 10102821

[B22] Kosslyn S. M. Thompson W. L. Kim I. J. Alpert N. M. (1995). Topographical representations of mental images in primary visual cortex. Nature 378, 496–498 10.1038/378496a0 7477406

[B23] Kumar T. Glaser D. (1993). Initial performance, learning, and observer variability for hyperacuity tasks. Vision Res. 33, 2287–2300 10.1016/0042-6989(93)90106-7 8273293

[B24] Lu Z. L. Dosher B. A. (2004). Perceptual learning retunes the perceptual template in foveal orientation identification. J. Vis. 4, 44–56 10.1167/4.8.265 14995898

[B25] Mast F. Kosslyn S. M. Berthoz A. (1999). Visual mental imagery interferes with allocentric orientation judgements. Neuroreport 10, 3549–3553 10.1097/00001756-199911260-00016 10619642

[B26] Mast F. W. Kosslyn S. M. (2002). Visual mental images can be ambiguous: insights from individual differences in spatial transformation abilities. Cognition 86, 57–70 10.1016/S0010-0277(02)00137-3 12208651

[B27] Pearson J. Tong F. (2011). Evaluating the mind’s eye: the metacognition of visual imagery. Psychol. Sci. 22, 1535–1542 10.1177/0956797611417134 22058106

[B28] Pourtois G. Rauss K. S. Vuilleumier P. Schwartz S. (2008). Effects of perceptual learning on primary visual cortex activity in humans. Vision Res. 48, 55–62 10.1016/j.visres.2007.10.027 18082238

[B29] Pylyshyn Z. W. (2002). Mental imagery: in search of a theory. Behav. Brain Sci. 25, 157–238 10.1017/S0140525X02000043 12744144

[B30] Pylyshyn Z. W. (2003). Return of the mental image: are there pictures in the brain? Trends Cogn. Sci. 7, 113–118 10.1016/S1364-6613(03)00003-2 12639692

[B31] Slotnick S. D. Thompson W. L. Kosslyn S. M. (2005). Visual mental imagery induces retinotopically organized activation of early visual areas. Cereb. Cortex 1570–1583 10.1093/cercor/bhi035 15689519

[B32] Sowden P. T. Rose D. Davies I. R. (2002). Perceptual learning of luminance contrast detection: specific for spatial frequency and retinal location but not orientation. Vision Res. 42, 1249–1258 10.1016/S0042-6989(02)00019-6 12044757

[B33] Tartaglia E. M. Bamert L. Herzog M. H. Mast F. W. (2012). Perceptual learning of motion discrimination by mental imagery. J. Vis. 12, 1–10 10.1167/12.5.1 22693332

[B34] Tartaglia E. M. Bamert L. Mast F. W. Herzog M. H. (2009). Human perceptual learning by mental imagery. Curr. Biol. 19, 2081–2085 10.1016/j.cub.2009.10.060 19962313

[B35] Vogels R. Orban G. A. (1985). The effect of practice on the oblique effect in line orientation judgements. Vision Res. 25, 1679–1687 10.1016/0042-6989(85)90140-3 3832592

